# Never and under cervical cancer screening in Switzerland and Belgium: trends and inequalities

**DOI:** 10.1186/s12889-020-09619-z

**Published:** 2020-10-07

**Authors:** Vladimir Jolidon, Vincent De Prez, Barbara Willems, Piet Bracke, Stéphane Cullati, Claudine Burton-Jeangros

**Affiliations:** 1 Institute of Sociological Research, University of Geneva, 40 Bd du Pont-d’Arve, 1211 Genève 4, Switzerland; 2 Department of Sociology, Ghent University, Korte Meer 5, 9000 Ghent, Belgium; 3 Population Health Laboratory, University of Fribourg, Rte des Arsenaux 41, 1700 Fribourg, Switzerland

## Abstract

**Background:**

Research on inequalities in cervical cancer screening (CCS) participation has overlooked the distinction between ‘never-’ and ‘under-screeners’ while different socioeconomic and demographic determinants may underlie ‘non-’ and ‘under-’ screening participation. This study examines socioeconomic and demographic inequalities in never and under CCS participation. We compare cross-national prevalence and trends among these two groups in Switzerland and Belgium, two countries with similar opportunistic CCS strategy but different healthcare systems.

**Methods:**

Data on 38,806 women aged 20–70 from the Swiss Health Interview Survey (1992–2012) and 19,019 women aged 25–64 from the Belgian Health Interview Survey (1997–2013), both population-based cross-sectional nationally representative surveys, was analysed. Weighted adjusted prevalence ratios were estimated with multivariate Poisson regressions.

**Results:**

Over the studied period, never screening prevalence was about 15% in both Switzerland and Belgium and under screening prevalence about 14.0%. Socioeconomic gradients were found among both never- and under-screeners. Higher income women had lower never and under screening prevalence in Switzerland and a similar gradient in education was observed in Belgium. Importantly, distinct socioeconomic and demographic determinants were found to underlie never and under screening participation. Never screening was significantly higher among foreign nationals in both countries and this association was not observed in under screening. Never screening prevalence was lower among older age groups, while under screening increased with older age. Over time, age inequalities diminished among never- and under- screeners in Switzerland while educational inequalities increased among never-screeners in Belgium.

**Conclusion:**

Findings revealed that determinants of screening inequalities differed among never- and under-screeners and hence these should be addressed with different public health strategies. Crucially, socioeconomic and demographic inequalities were more pronounced among never-screeners who appeared to face more structural and persistent inequalities. Differences between the two countries should also be noted. The more liberal-type Swiss healthcare systems appeared to shape income-related screening inequalities, while education appeared to be a stronger determinant of never- and under-screening in Belgium.

## Introduction

Cervical cancer (CC) ranks fourth worldwide for both incidence and mortality [1]. Europe’s overall incidence rate is 11.2 per 100,000 women per year, in age-standardised rate by world population, and Switzerland and Belgium’s incidence rates are lower than the European average with 3.8 and 7.8 per 100,000, respectively [1]. CC is the cancer that can most effectively be prevented by screening. It was shown that cervical cancer screening (CCS), and particularly organised population-based CCS, reduces both incidence and mortality [2–4]. CCS may have reduced CC incidence and mortality by 80% in different contexts [5]. The beneficial effects of CCS are reflected in the low CC incidence and mortality rates of countries which introduced effective CCS. For example, Western Europe has an average CC incidence rate of 6.8 per 100,000 women, whereas that of Central and Eastern Europe, characterised by lower screening coverage, is of 16.0 per 100,000 [6]. Hence, European and international guidelines recommend that CCS should be organised and population-based [5, 7].

Socioeconomic and demographic factors such as education, income, age, nationality and marital status were shown to be associated with adherence to CCS, and that such disparities persisted over time [8–12]. However, to our knowledge, few studies analysed the distinct social and demographic characteristics of ‘never-’ and ‘under-’ screeners (those who never screened and those who did screen but not within the 3-year recommendation period) and previous research on CCS in Switzerland and Belgium focused on women who screened within the 3-year recommended period [11, 13].

This study addresses ‘never’ and ‘under’ CCS participation in Switzerland and Belgium using cross-sectional data from the Swiss and Belgian national health interview surveys spanning from 1992 to 2013. Comparing two countries with similar CCS strategy but different healthcare systems appeared to be particularly relevant. That is, neither country has implemented CCS programmes (although Belgium’s Flemish region started a CCS programme since 2013 [14]) and hence rely on opportunistic CCS. As opposed to organised CCS, opportunistic CCS leans on the individual’s awareness and initiative to screen and on the doctor’s screening recommendation to their patients. In both countries, it is mainly the gynaecologist who recommends a CCS to women during routine examinations [15, 16]. A cross-national perspective on CCS is also relevant since most cancer screening studies tended to focus on specific countries [11, 14, 17].

The present study distinguishes between ‘never-’ and ‘under-screeners’ and hypothesises that these two groups are affected by different socioeconomic determinants of cancer screening participation. In contexts of opportunistic CCS, we expect to find never and under screening inequalities to persist over time in both Switzerland and Belgium. We also expect to find different patterns and trends of inequalities in CCS participation in the two countries since these vary across contexts and are embedded in health systems. In sum, this study aims to compare the prevalence and trends of never and under CCS in Switzerland and Belgium and investigate socioeconomic and demographic inequalities and the trend of those inequalities over time.

## Methods

Our study is based on data collected by the 1992–2012 Swiss Health Interview Survey (SHIS) and the 1997–2013 Belgian Health Interview Survey (BHIS), both nationally representative cross-sectional surveys comprising five waves and based on a stratified random selection of residents older than 14 years of age. The former was implemented in 1992, 1997, 2002, 2007 and 2012, and the latter in 1997, 2001, 2004, 2008 and 2013. The SHIS study sample included women aged 20 to 70 years old (*N* = 38,806) and that of the BHIS included women aged 25 to 64 years old (*N* = 19,019), based on each country’s CCS age recommendation. After excluding respondents who had missing data on the outcome variable, predictors of interest and covariates, and had received cancer treatment/ diagnosis in the past 12 months, 31,800 women were included in the SHIS final sample, and 9442 in the BHIS sample.

### National contexts

The Swiss Law on Health Insurance mandated private health insurances to reimburse one CCS every 3 years for women from 18 to 69 years old since 1996 and the Swiss medical guidelines advised to perform the Pap smear test every 3 years on women aged 21–70 years [18]. The Belgian cervical cancer screening policy followed the European guidelines and recommended one CCS every 3 years to 25–64 years old women [14].

### Dependent and independent variables

Both the SHIS and the BHIS asked women if they ‘ever had a Pap smear’ (yes, no) and, if they answered ‘yes’, when was the last time they had it. The SHIS asked to provide the month and year of the last Pap smear, while the BHIS asked if the Pap smear was undertaken, ‘within past 12 months, more than 1 year but not more than 2 years ago, more than 2 years but not more than 3 years ago, more than 3 years but not more than 5 years ago, and not within the past 5 years’. We computed two binary dependent variables to analyse women who ‘never-screened’ (0 = ever screened, 1 = never screened), and women who ‘under-screened’ (0 = screened within the past 3 years, 1 = screened more than 3 years ago). Predictors of interest and covariates were selected based on their potential association with CCS [9, 11, 16, 19] and their availability in both the SHIS and BHIS. The following predictors of interest were analysed: education (primary, upper secondary, tertiary), monthly household income (1st to 5th quintile), employment status (employed, non-employed), partnership status (living, not living with a partner/ spouse), nationality (national citizen, foreign national), area of residence (urban, rural), and age ranges (20–29, 30–39 …). Educational levels followed the International Standard Classification of Education 2011 [20]. Household income was weighted according to the OECD-modified scale in both the SHIS and BHIS (based on the number of adults and children living in the household). Women who were unemployed, at home, retired and out of the labour force were all grouped in the non-employed category. Different age ranges were applied to the SHIS and BHIS samples since CCS recommendations differed in the two countries (Switzerland 20 to 70 and Belgium 25 to 64 years old). To control for potential associations with CCS screening, we included the following covariates in our analysis: self-rated health (very good, good, fair, bad, very bad), self-reported body mass index (underweight < 18.5 kg/m2, normal weight 18.5 to < 25, overweight & obese 25 to ≥30), a doctor visit (general practitioner or any specialist) in the last 12 months (yes, no) and currently smoking (yes, no). In the BHIS, the “doctor visit in the last 12 months” included a dentist visit whereas it did not in the SHIS.

### Statistical analysis

In order to evaluate inequalities in never and under CCS, adjusted prevalence ratios (APR) with 95% confidence intervals (CI) were estimated with Poisson regression models and robust variance estimators. Such models were shown to be adequate to analyse binary outcomes, particularly with cross-sectional data, and easier to interpret and communicate with prevalence terms as the measure of association [21, 22]. The two dependent variables were analysed in separate models and treated as binary variables. Models presented in Table 2 and Tables S.3a and S.3b (supplementary materials) were adjusted for all the independent variables mentioned above. Models presented in Table 2 analysed data from pooled waves and were also adjusted for time (survey wave variable). In order to evaluate the potential time trend of our predictors of interest, we performed one separate multivariate model for each of these with an interaction term between the predictor and the survey wave variable, along with all independent variables. The *P*-values of the interaction terms for time trend were reported in Table 2. Descriptive statistics and regression analyses were weighted for survey sampling and performed with SPSS 25 and STATA 14. SHIS data were also weighted for non-response bias, as detailed elsewhere [23]. Collinearity between independent variables was tested with variance inflation factors and did not reveal any potential collinearity. SHIS and BHIS analysis were performed separately by the authors of this study and results were subsequently compared and discussed.

## Results

### Participants’ characteristics

Swiss and Belgian samples are summarised in Table 1. Some differences between the two samples were partly due to the different age ranges applied; for example, in the Swiss sample, less women achieved primary and tertiary education and less women were in the first and second household income quintiles compared to the Belgian sample. In the Swiss sample, there was a higher proportion of underweight women and foreign nationals, and a lower proportion of women visited a doctor in the last 12 months, compared to the Belgian sample.

**Table 1:** Characteristics of eligible women in Switzerland (SHIS 1992–2012) and Belgium (BHIS 1997–2013) samples^a^

	Switzerland (women aged 20–70) *N* = 31,800		Belgium (women aged 25–64) *N* = 9442	
	N	%	N	%
*Main independent variables*				
Education				
Primary & lower secondary	4716	15	2820	28.8
Upper secondary	21,070	66.7	2925	32.6
Tertiary	6014	18.4	3697	38.6
Monthly household income				
1st quintile	7121	23.6	1723	16.2
2nd quintile	6233	20.9	1505	16
3rd quintile	6870	20.6	1776	19.4
4th quintile	6949	21.2	2061	23.1
5th quintile	4627	13.7	2377	25.3
Employment				
Employed	20,611	64.9	5884	63.7
Unemployed/ non-employed	11,189	35.1	3558	36.3
Partnership status				
Single, widow, divorced and separated (and dissolved partnership)	11,830	28.7	2759	24.1
Living with spouse/ partner	19,970	71.3	6683	75.9
Age				
20–29 (CH)/ 25–29 (BE)	5097	18.7	1150	11.5
30–39	7593	24.1	2682	29.2
40–49	7066	23.4	2479	27.2
50–59	6025	17.9	2172	22.7
60–70 (CH)/ 60–64 (BE)	6019	15.9	959	9.4
Nationality				
National citizen (Swiss / Belgian)	27,653	81.7	8493	93.2
Foreign national	4147	18.3	949	6.8
Area of residence				
Urban	22,470	72.1	6988	72.4
Rural	9330	27.9	2454	27.6
*Covariates*				
Self-rated health				
Very good	9163	29.1	2403	25.9
Good	18,165	57.5	4833	52.6
Fair	3514	10.6	1798	17.9
Bad	808	2.3	339	3.1
Very bad	150	0.4	69	0.5
BMI				
Normal weight	18,734	59.3	5538	58.4
Underweight	4502	14.2	425	4.3
Overweight & obesity	8564	26.6	3479	37.3
Doctor visit in the last 12 months				
No	5117	16.2	1037	10.6
Yes	26,683	83.8	8405	89.4
Smoking				
No	22,582	71.8	6868	74.3
Yes	9218	28.2	2574	25.7
*Dependent variables*				
Never had a CCS				
Never	5093	15.8	1526	15.0
Ever	26,707	84.2	7916	85.0
Under-screening (subsample: CH: *N* = 25,680; BE: *N* = 7788)				
Under-screening (overdue CCS)	3581	13.0	1124	14.7
Up-to-date CCS (within the past 3 years)	22,099	87.0	6664	85.3

^a^Proportions are weighted for sampling strategy in the SHIS and BHIS, and also for non-response in the SHIS

*SHIS* Swiss Health Interview Survey, *BHIS* Belgian Health Interview Survey

### Prevalence and trends in never and under cervical cancer screening

**Fig. 1:**
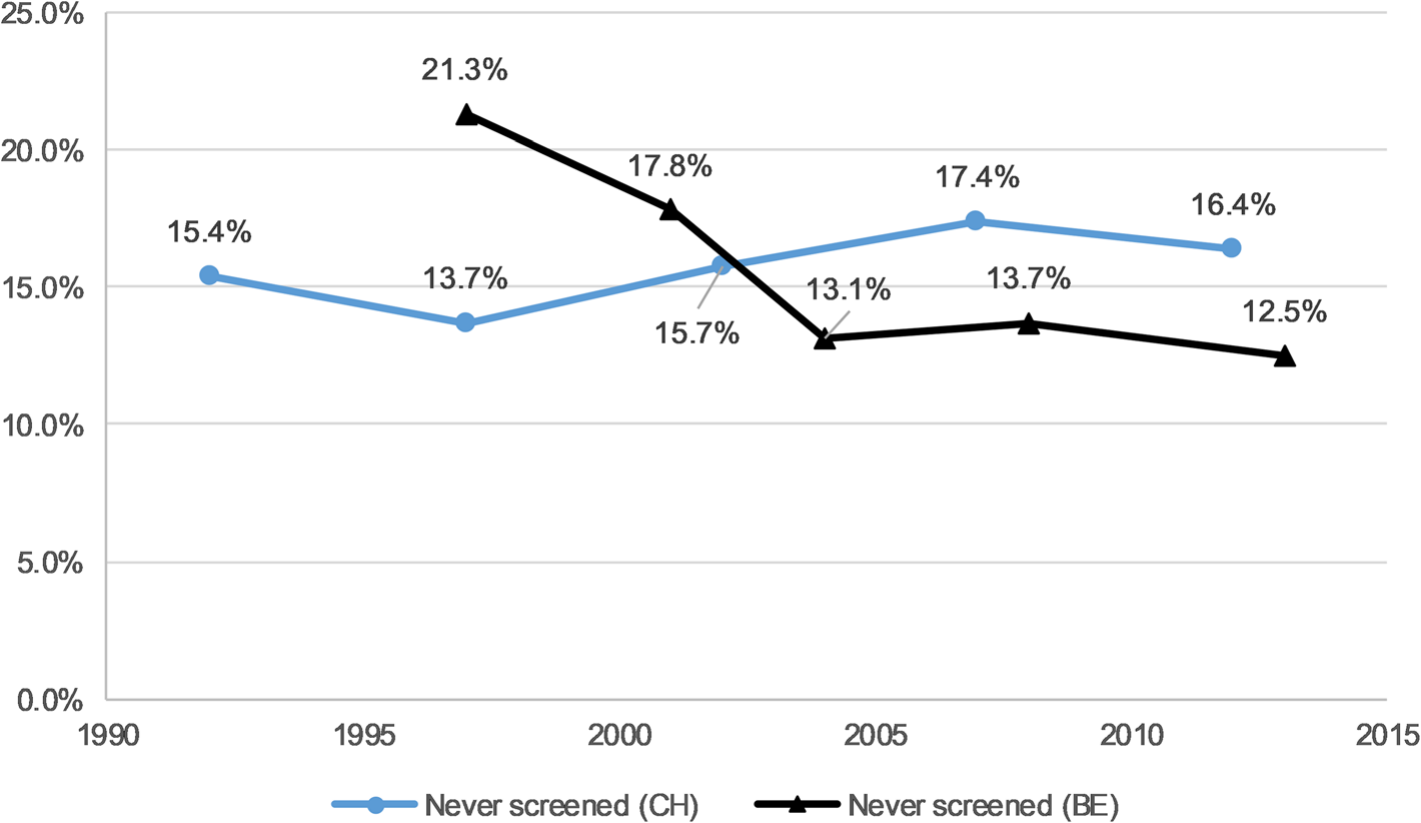
“Never had a CCS” weighted prevalence among eligible women in Switzerland and Belgium^1^. ^1^ Swiss Health Interview Survey (SHIS) 1992–2012 and Belgian Health Interview Survey (BHIS) 1997–2013; Adjusted APR for time: CH = 95%CI 1.04–1.09, and BE = 95%CI 0.84–0.92 (see Table 2). Notes: CH = Switzerland; BE = Belgium

**Fig. 2:**
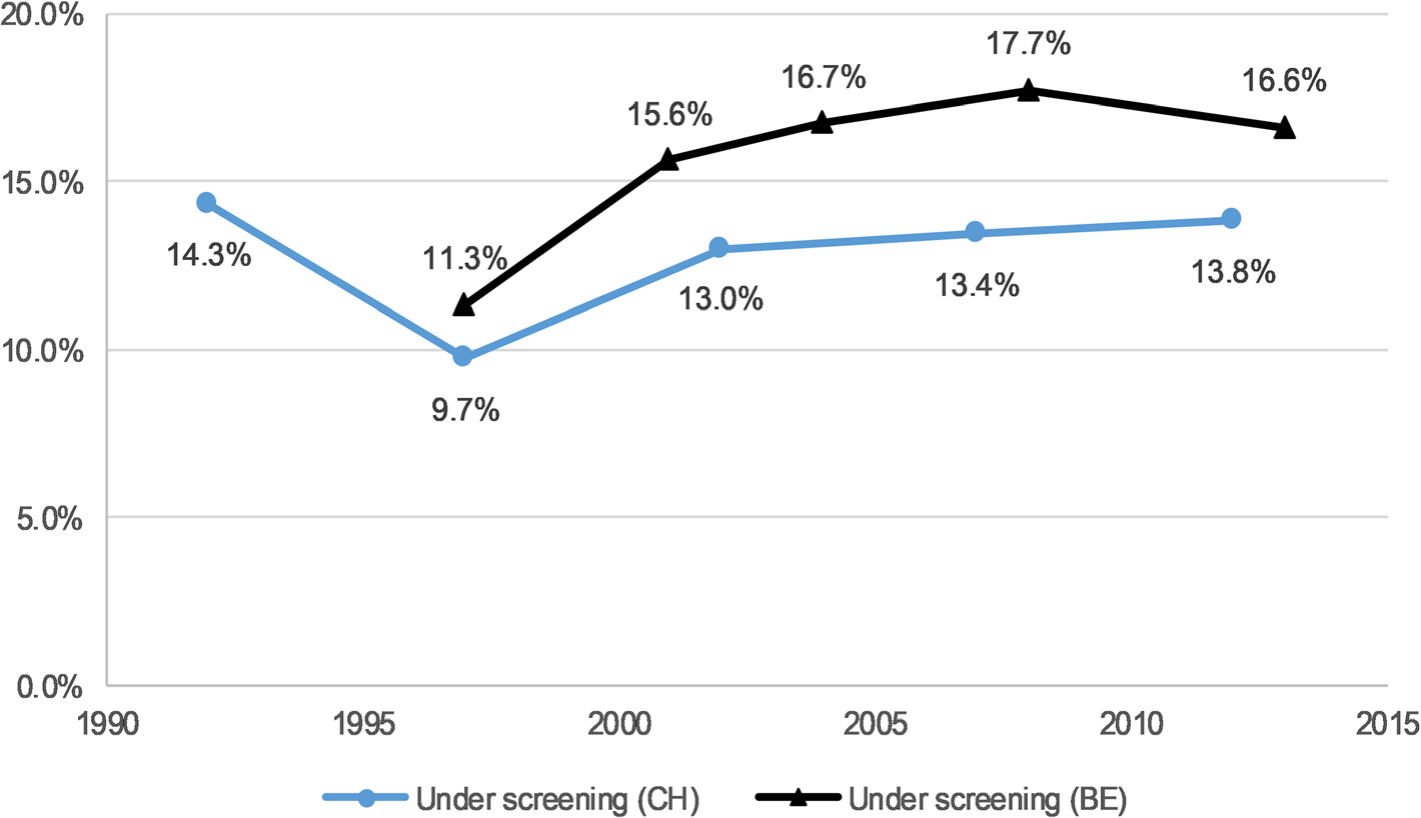
“Under-screening” weighted prevalence among eligible women in Switzerland and Belgium^1^. ^1^ Swiss Health Interview Survey (SHIS) 1992–2012 and Belgian Health Interview Survey (BHIS) 1997–2013; Adjusted APR for time: CH = 95%CI 0.98–1.04, and BE = 95%CI 1.02–1.13 (see Table 2). Notes: CH = Switzerland; BE = Belgium

Over the studied period, never CCS prevalence was 15.8% in Switzerland and 15.0% in Belgium (Table 1). The prevalence increased in Switzerland by 6% (APR 1.06, 95%CI 1.04–1.09) and decreased in Belgium by 12% on average by survey wave (APR 0.88, 95%CI 0.84–0.92) (Table 2). Under CCS prevalence was 13.0% in Switzerland and 14.7% in Belgium. It increased in Belgium by 7% (APR 1.07, 95%CI 1.02–1.13) in average by survey wave and did not show a significant tendency in Switzerland throughout the studied period (APR 1.01, 95%CI 0.98–1.04). Figures 1 and 2 show the prevalence of never and under screening over time for both Switzerland and Belgium.

**Table 2:** Adjusted prevalence rations (APR) for never and under CCS among eligible women in Switzerland and Belgium^a^

	Never had a CCS in Switzerland (women aged 20–70) *N* = 31,800			Never had a CCS in Belgium (women aged 25–64) N = 9442			CC under-screening in Switzerland (women aged 20–70) *N* = 25,680			CC under-screening in Belgium (women aged 25–64) *N* = 7788		
	APR	95% CI	*P-*values for trend over 5 waves^b^	APR	95% CI	*P-*values for trend over 5 waves^a^	APR	95% CI	*P-*values for trend over 5 waves^b^	APR	95% CI	*P-*values for trend over 5 waves^b^
Education (ref: primary & lower secondary)			0.723			0.018*			0.19			0.712
Upper secondary	0.66	0.61–0.72		0.80	0.68–0.93		0.90	0.81–0.99		0.84	0.71–0.99	
Tertiary	0.66	0.59–0.74		0.59	0.49–0.70		0.90	0.79–1.03		0.69	0.56–0.85	
Employment (ref: employed)			0.162			0.001**			0.48			0.834
Unemployed/ non-employed	0.97	0.90–1.04		1.15	0.97–1.35		1.03	0.95–1.13		0.98	0.83–1.17	
Monthly household income (ref: 1st quintile)			0.214			0.087			0.024*			0.949
2nd quintile	0.81	0.74–0.89		0.74	0.62–0.89		0.98	0.88–1.10		0.87	0.69–1.08	
3rd quintile	0.82	0.75–0.90		0.73	0.60–0.89		0.90	0.81–1.00		0.86	0.69–1.07	
4th quintile	0.78	0.71–0.86		0.63	0.51–0.78		0.84	0.75–0.94		0.92	0.73–1.16	
5th quintile	0.75	0.67–0.84		0.62	0.49–0.79		0.80	0.70–0.92		0.74	0.57–0.95	
Partnership status (ref: no partner)			0.032*			0.126			0.994			0.843
Living with spouse/ partner (living in couple)	0.71	0.66–0.76		0.80	0.70–0.92		0.82	0.76–0.88		0.86	0.73–1.00	
Age (ref: CH: 20–29 / BE: 25–29)			0.033*			0.977			0.004**			0.524
30–39	0.54	0.49–0.58		0.62	0.53–0.74		1.57	1.27–1.95		1.96	1.28–3.01	
40–49	0.38	0.35–0.42		0.50	0.42–0.60		2.70	2.20–3.30		2.56	1.69–3.88	
50–59	0.39	0.35–0.44		0.47	0.38–0.58		3.95	3.23–4.82		3.26	2.14–4.97	
CH: 60–70 / BE: 60–64	0.56	0.50–0.62		0.55	0.43–0.70		6.49	5.31–7.92		5.18	3.34–8.04	
Nationality (ref: national citizen)			0.368			0.048*			0.598			0.557
Foreign national	1.65	1.53–1.78		1.54	1.30–1.81		0.91	0.80–1.03		1.15	0.86–1.53	
Area of residence (ref: urban)			0.88			0.032*			0.705			0.329
Rural	1.14	1.07–1.22		0.99	0.86–1.14		1.19	1.11–1.29		1.03	0.89–1.20	
SHIS / BHIS waves (ref: wave 1)	1.06	1.04–1.09		0.88	0.84–0.92		1.01	0.98–1.04		1.07	1.02–1.13	

*APR* Adjusted prevalence ratios. APR are weighted for sampling strategy in the SHIS and BHIS, and also for non-response in the SHIS. Variables used for adjustment: self-rated health, body mass index, doctor visit in the last 12 months, smoking

* = *p* < 0.05; ** = *p* < 0.01; *** = *p* < 0.001

^a^ SHIS 1992–2012 and BHIS 1997–2013

^b^ *P-*values for trend were estimated separately for each predictor of interest with multivariate models including the interaction term (between the predictor of interest and the survey wave variable)

### Inequalities and trends in never and under cervical cancer screening

#### Never screening

In both Switzerland and Belgium, never screening prevalence was lower among women with higher levels of education, higher household incomes, living in couple and from older age groups (Table 2). Prevalence was higher among foreign nationals. In Switzerland, never screening prevalence followed a gradient from the 3rd to 5th household income quintiles, i.e. the higher income, the less women never screened. Never screening was higher among women living in a rural area. As showed by the time interaction term, prevalence significantly fluctuated over the 5 waves for partnership status however no clear trend was observed (Table S.3a). The time interaction term for age was also significant and the difference between the APRs of the 20–29 year-old women and older age groups reduced from 1992 to 2012. In Belgium, never screening prevalences followed an education and income gradient as these diminished among more educated and higher income women (Table 2). Nationality and area of residence had a significant time interaction term but did not show a clear trend (Table S.3a). Education inequality also varied significantly throughout the period, APRs were significant in 1997, 2008 and 2013 and the differences between educational strata slightly increased in 2013. Non-employment also had a significant time trend. At the beginning of the period (1997–2001), non-employed women had a lower never screening prevalence compared to employed women (although APRs were not significant, see Table S.3a, supplementary materials), and this tendency reversed from 2004 to 2013 with non-employment increasing never screening prevalence.

#### Under screening

In both Switzerland and Belgium, under screening prevalence was significantly associated and diminished with 5th household income quintiles, upper secondary education and living in couple (Table 2). Under screening prevalence increased with the increase of age. In Switzerland, under screening prevalence significantly decreased following a gradient throughout the 3rd, 4th and 5th household income quintiles. Under screening prevalence increased with rural area of residence. Time fluctuation for household income and age groups was significant. Household income did not show a clear trend and age groups showed a tendency as inequalities between women aged 20–29 and older age groups reduced over time (see Table S.3b, supplementary materials). In Belgium, under screening prevalence followed a gradient in education and significantly decreased from upper secondary to tertiary education level. Predictors of interest did not change over the studied period since time interactions were not significant.

## Discussion

This study examined trends of never and under CCS prevalence, and socioeconomic and demographic inequalities, using five waves of the SHIS and BHIS. Over the studied period, both countries had similar prevalences, about 15% of never screening and 14% of under screening. Although these levels of ‘never’ and ‘under’ CCS participation are relatively low compared to other European countries [24], socioeconomic inequalities were observed as women with higher education and income showed lower never screening prevalence in both countries. These inequalities resembled those revealed in other screening tests in other countries which also relied on opportunistic screening [8, 25, 26]. The higher participation in CCS of highly educated women could be explained by their higher ‘health literacy’, a more future-oriented attitude and better risk perception which favour more preventive-focused health decision [27]. Additionally, negative attitudes towards cancer screening among lower socioeconomic participants could also be mediating the association between income and screening participation [17].

Belgium showed a gradient of screening inequalities in education and income levels among never-screeners while this gradient was only evident for income in Switzerland. We may advance that inequalities were shaped by economic determinants in the more liberal-type Swiss healthcare system. Although health insurance is compulsory for all Swiss residents, patient’s healthcare cost participation and high out-of-pocket payments cause high healthcare forgoing particularly among those with lower incomes [28]. The Swiss healthcare system shortcomings in addressing health inequalities and implementing more health prevention were already identified and partly attributed to the fragmented nature of the Swiss public health system (with high autonomy of the cantons), which remains an obstacle to coordinate healthcare at the national level [29]. This income gradient of screening inequalities persisted among under-screeners in Switzerland suggesting that lower income women might be foregoing preventive healthcare. As a qualitative study in Switzerland pointed out, women who faced financial hardship either perceived the screening cost to be an “issue” or an “unnecessary expense”, particularly if they considered themselves to be “in good health” [30].

In both countries, education and income inequalities seemed to be less pronounced among under-screeners compared to never-screeners. We hypothesise that women who screened at least once were more acquainted with prevention and screening and hence less affected by socioeconomic barriers to screening. Among under-screeners, more practical issues might constitute barriers to screening, such as scheduling a doctor’s appointment during the working week. Conversely, among never-screeners, socioeconomic barriers to undertake a very first screening appeared to be stronger and more persistent over time.

Older age reduced never screening prevalence in both countries and so did living in couple. Women who are in a partnership, and older women, are more likely to visit a gynaecologist, either to conceive or for contraception, and we may expect them to undertake CCS at least once, as they enter their reproductive period [11]. On the other hand, our results interestingly revealed that under screening increased among older women in both countries. We may advance that, as they become older, women start neglecting their routine screening, particularly after repeated negative screens, or their doctors may insist less on screening within the recommended time. Qualitative studies suggested that CCS participation declines as women enter a life stage in which sexuality and pregnancy are less central and visits to the gynaecologist less frequent [30]. Older women are also more likely to cite lower levels of concern with CC (or lower perceived risk) and more likely to express embarrassment and fear of pain [31].

Based on our results, we suggest that never- and under-screeners should be addressed with different strategies. A study on CCS uptake in the United Kingdom stressed that policy interventions should consider the CCS non-participants’ heterogeneity of motivations and attitudes. It showed that 51% of CCS non-participants “intended to screen but were overdue” since they failed to translate intention into action, and 28% were unaware of screening [32]. Younger and more disadvantaged women were more likely to be found among those groups. We suggest that measures to reduce inequalities in CCS should focus on never-screeners – with an effort to tackle issues such as screening awareness among the most disadvantaged – while interventions among under-screeners should pay attention to the intention-behaviour gap in order to improve participation (for example, through reminders), access and practical issues (such as in scheduling a doctor’s appointment), although inequalities among under-screeners should not be neglected.

In both countries, screening inequality between nationals and foreigners was found among never-screeners, although not among under-screeners which supports our hypothesis that sociodemographic inequalities were stronger among never-screeners. Living in rural area increased both never and under screening prevalence in Switzerland, while such inequalities were not found in Belgium. This is consistent with studies of other cancer screening tests in Switzerland [33] which pointed out that women living in a rural area might under-screen while their urban counterparts are more likely to over-screen [34, 35].

Over the studied period, never CCS prevalence increased 6% in average by survey wave in Switzerland while it decreased 12% by survey wave in Belgium. Under screening did not show a clear tendency in Switzerland and it increased 7% by survey wave in Belgium. In Switzerland, Burton-Jeangros et al. [11] observed a slight decrease of CCS prevalence, based on the same dataset of the present study. Our results suggested that this decrease stemmed from a slight increase of never screening – rather than under screening – over the same period. In Belgium, an invitation programme was in place in Flanders from the mid 1990’s until the early 2000’s and could have contributed to reducing never screening. Nevertheless, no impact of sending invitations on screening uptake was found [14].

Inequalities between age groups among both never-screeners and under-screeners diminished in Switzerland throughout the studied period. This may have been caused by a cohort effect, i.e. by a generation of women who started to screen more at a younger age than the previous generation, and continued to do so as they became older. In Belgium, we observed increasing education-based inequalities among never-screeners over time which were explained in previous studies by a combination of under-screening among women with lower education and over-screening among women with higher education in a context of opportunistic screening [13].

Reliance on opportunistic screening in Switzerland and Belgium may have contributed to the persistence of the CCS inequalities observed in our study. Studies showed that opportunistic screening entailed higher screening inequalities, inconsistent quality and inefficiencies such as over-screening [8, 36–38]. As a Swiss-Belgian comparative study on screening overuse showed, although declining, over-screening is persistent in both countries [35]. An organised approach to CCS, with quality assurance framework and strategies to improve never and under-screeners’ participation, may minimise the adverse effects of unequal screening and maximise benefits from a public health and cost perspective [37, 39]. In Switzerland, a nation-wide CCS programme would help tackle screening inequalities in the context of a fragmented healthcare system which contributes to reproducing health inequalities. In Belgium, the CCS organised programme which was launched in Flanders should be extended nationwide to avoid reinforcing regional inequalities.

Limitations of our study are worth noting. The SHIS and BHIS are cross-sectional surveys hence our results do not measure whether individual respondents complied or not across time with the 3-year recommended screening interval. However, from an aggregated (population) level perspective, our ‘under-screening’ variable allowed us to account for the women who screened ‘more than 3 years ago’ (the ‘under-screeners’), as opposed to those who screened within the recommended interval. We could not control for related preventive practices of HPV vaccination and HPV testing in our models since this information was not available in the SHIS and the BHIS (only the BHIS 2013 wave collected information on HPV vaccine uptake). HPV vaccination programmes were implemented among teenagers and young women in 2008 and 2010 in Switzerland and Belgium respectively (2011 in the French-speaking part of Belgium) [40, 41]. Consequently, these could only have affected the CCS practices of the youngest cohort of the SHIS’ and BHIS’ last wave (notably, only 25 women aged 25–49 had the vaccine in the BHIS 2013 wave) and, hence, influenced our results in a negligible way. Regarding HPV testing, this test has a 5-year recommended screening interval which is larger than the one of Pap smear. GPs and gynaecologists could have offered this test as an alternative to Pap smear which may have affected our under-screening measure. Nonetheless, CCS recommendation guidelines were based on Pap smear as primary screening in both countries during the studied period [14, 18]. Gynaecologists implemented Pap smear as part of the routine check-up in both countries and HPV testing was not reimbursed by the health insurance in Switzerland, and only partly in Belgium [14, 15, 42–44]. The effects of HPV vaccination and testing on CCS practices are difficult to evaluate. However, our data did not show an increase of under screening which would suggest that new preventive techniques had supplanted Pap smear since 2008 in either country. We cannot account for the complexity of national contexts and healthcare systems. The healthcare system design (public and private mix), levels of public health expenditures, density of general practitioners, payment schemes for general practitioners and specialists, insurance coverage, amount of private out-of-pocket payments, accessibility of care, as well as cultural and environmental factors, affect screening participation and inequalities and may produce confounding despite adjustments [12, 45, 46]. Self-reported CCS data may be affected by recall bias. Studies showed that women fairly correctly reported CCS uptake, however, they tend to report their last CCS more recently than it actually took place - a phenomenon described as “telescoping” - which may cause over-reporting of screening within a specific timeframe and hence underestimation of under-screening [47, 48]. Women may also over-report CCS by recalling a routine gynaecologic exam without CCS as including a CCS [47]. Additionally, social desirability bias may lead to underestimates of never and under participation or time since last screening test [47] and response bias might also affect the data since women with higher education level tend to report screening participation more often [49]. In spite of the SHIS and BHIS representativeness limitations, the use of the weighting factors allowed for inference from the sample to the total population of Switzerland and Belgium. Finally, further research is needed to inquire the motivations and attitudes which lie behind ‘never’ or ‘under’ screening participation in order to design policy interventions.

## Conclusion

Screening inequalities among never- and under-screeners persisted over time in both Switzerland and Belgium and socioeconomic and demographic determinants of screening inequalities differed between these groups. Inequalities appeared to be more pronounced amongst never-screeners compared to under-screeners hence results stressed that the two groups should be addressed with different strategies. Differences were highlighted between the two countries. Inequalities appeared to be shaped by economic determinants in the more liberal-type Swiss healthcare system, as showed by the income gradient among never- and under-screeners, while inequalities followed an education gradient in Belgium. Finally, both Switzerland and Belgium could benefit from an organised approach to CCS in order to mitigate the screening inequalities observed in our study and improve efficiency from a public health perspective.

## Supplementary information

**Additional file 1 **: **Table S.3a**. Adjusted prevalence rations (APR) for never CCS among eligible women in Switzerland and Belgium^a^. **Table S.3b**. Adjusted prevalence rations (APR) for under CCS among eligible women in Switzerland and Belgium^a^.

## Data Availability

This study used the data from the Swiss Health Interview Survey and the Belgium Health Interview Survey. The Swiss data are available for fee (1600 Swiss Francs, plus 7.7% tax) and users must request permission from the Swiss Federal Statistical Office. The Belgian data are available for researchers, but according to the Belgian legislation an authorisation has to be obtained from the Belgian Commission for the Protection of Privacy.

## Abbreviations

CC: Cervical cancer
CCS: Cervical cancer screening
SHIS: Swiss Health Interview Survey
BHIS: Belgian Health Interview Survey
CH: Switzerland
BE: Belgium
APR: Adjusted prevalence ratio
