# Supplementary material for: Never and under cervical cancer screening in Switzerland and Belgium: trends and inequalities
Source: BMC Public Health. 2020 Oct 7;20:1517. doi: 10.1186/s12889-020-09619-z (PMC7542418; doi:10.1186/s12889-020-09619-z)
Supplement: Supplementary file 1 — Additional file 1 : Table S.3a. Adjusted prevalence rations (APR) for never CCS among eligible women in Switzerland and Belgiuma. Table S.3b. Adjusted prevalence rations (APR) for under CCS among eligible women in Switzerland and Belgiuma. [file 12889_2020_9619_MOESM1_ESM.docx]

**Supplementary materials**

| **Table S.3a. Adjusted prevalence rations (APR) for never CCS among eligible women in Switzerland and Belgium^a^** | | | | | | | | | | | | | | | | | | | | |
| --- | --- | --- | --- | --- | --- | --- | --- | --- | --- | --- | --- | --- | --- | --- | --- | --- | --- | --- | --- | --- |
|  | Never had a CCS in Switzerland (women aged 20-70) | | | | | | | | | | Never had a CCS in Belgium (women aged 25-64 y/o) | | | | | | | | | |
|  | 1992 N = 5452 | | 1997 N = 3838 | | 2002 N = 7495 | | 2007 N = 6845 | | 2012 N = 8170 | | 1997 N = 2114 | | 2001 N = 2073 | | 2004 N = 1943 | | 2008 N = 1664 | | 2013 N = 1648 | |
|  | APR | 95% CI | APR | 95% CI | APR | 95% CI | APR | 95% CI | APR | 95% CI | APR | 95% CI | APR | 95% CI | APR | 95% CI | APR | 95% CI | APR | 95% CI |
| Education (ref: primary & lower secondary) |  |  |  |  |  |  |  |  |  |  |  |  |  |  |  |  |  |  |  |  |
| Upper secondary | 0.7 | 0.59-0.82 | 0.58 | 0.46-0.72 | 0.71 | 0.59-0.85 | 0.61 | 0.50-0.74 | 0.63 | 0.53-0.75 | 0.76 | 0.57-1.00 | 0.97 | 0.72-1.29 | 0.89 | 0.62-1.29 | 0.68 | 0.48-0.98 | 0.62 | 0.40-0.94 |
| Tertiary | 0.77 | 0.61-0.98 | 0.68 | 0.47-0.97 | 0.62 | 0.47-0.81 | 0.61 | 0.48-0.77 | 0.58 | 0.47-0.72 | 0.57 | 0.42-0.78 | 0.75 | 0.54-1.05 | 0.71 | 0.43-1.16 | 0.47 | 0.30-0.75 | 0.41 | 0.25-0.69 |
| Employment (ref: employed) |  |  |  |  |  |  |  |  |  |  |  |  |  |  |  |  |  |  |  |  |
| Unemployed/ non-employed | 0.76 | 0.64-0.91 | 0.86 | 0.68-1.08 | 1.13 | 0.97-1.32 | 1.15 | 0.99-1.34 | 1.01 | 0.86-1.19 | 0.97 | 0.75-1.25 | 0.81 | 0.60-1.08 | 1.29 | 0.88-1.90 | 1.40 | 0.90-2.19 | 1.77 | 1.15-2.72 |
| Monthly household income (ref: 1st quintile) |  |  |  |  |  |  |  |  |  |  |  |  |  |  |  |  |  |  |  |  |
| 2^nd^ quintile | 0.79 | 0.63-0.99 | 0.94 | 0.74-1.20 | 0.75 | 0.62-0.92 | 1.02 | 0.85-1.23 | 0.79 | 0.66-0.96 | 0.81 | 0.60-1.10 | 0.73 | 0.50-1.07 | 0.65 | 0.41-1.04 | 0.87 | 0.56-1.35 | 0.73 | 0.40-1.33 |
| 3^rd^ quintile | 0.67 | 0.55-0.81 | 0.7 | 0.52-0.94 | 0.92 | 0.76-1.11 | 1.04 | 0.86-1.25 | 0.84 | 0.69-1.01 | 0.65 | 0.48-0.89 | 0.70 | 0.49-1.01 | 0.47 | 0.29-0.75 | 0.95 | 0.56-1.59 | 1.23 | 0.75-2.00 |
| 4^th^ quintile | 0.69 | 0.56-0.85 | 0.78 | 0.59-1.04 | 0.82 | 0.66-1.01 | 0.95 | 0.77-1.17 | 0.84 | 0.69-1.03 | 0.73 | 0.51-1.06 | 0.64 | 0.44-0.94 | 0.35 | 0.19-0.62 | 0.57 | 0.31-1.06 | 1.02 | 0.58-1.79 |
| 5^th^ quintile | 0.58 | 0.40-0.84 | 0.75 | 0.53-1.06 | 0.9 | 0.72-1.13 | 0.86 | 0.69-1.08 | 0.74 | 0.57-0.95 | 0.80 | 0.53-1.20 | 0.52 | 0.34-0.80 | 0.42 | 0.24-0.74 | 0.72 | 0.38-1.37 | 0.77 | 0.41-1.46 |
| Partnership status (ref: no partner) |  |  |  |  |  |  |  |  |  |  |  |  |  |  |  |  |  |  |  |  |
| Living with spouse/ partner | 0.66 | 0.56-0.77 | 0.7 | 0.58-0.84 | 0.65 | 0.57-0.74 | 0.85 | 0.74-0.97 | 0.63 | 0.55-0.71 | 0.65 | 0.51-0.83 | 0.81 | 0.63-1.04 | 0.99 | 0.71-1.40 | 0.88 | 0.64-1.22 | 0.83 | 0.58-1.21 |
| Age (ref: 20-29 / BE: 25-29) |  |  |  |  |  |  |  |  |  |  |  |  |  |  |  |  |  |  |  |  |
| 30-39 | 0.42 | 0.34-0.52 | 0.41 | 0.32-0.51 | 0.6 | 0.49-0.72 | 0.58 | 0.50-0.69 | 0.70 | 0.58-0.85 | 0.61 | 0.45-0.83 | 0.58 | 0.42-0.79 | 0.56 | 0.37-0.86 | 0.75 | 0.47-1.19 | 0.64 | 0.39-1.06 |
| 40-49 | 0.35 | 0.27-0.45 | 0.29 | 0.21-0.41 | 0.41 | 0.33-0.51 | 0.33 | 0.27-0.40 | 0.54 | 0.45-0.65 | 0.67 | 0.49-0.93 | 0.36 | 0.26-0.51 | 0.40 | 0.27-0.61 | 0.56 | 0.34-0.92 | 0.49 | 0.30-0.81 |
| 50-59 | 0.48 | 0.37-0.62 | 0.30 | 0.21-0.42 | 0.43 | 0.35-0.53 | 0.32 | 0.25-0.41 | 0.44 | 0.36-0.55 | 0.47 | 0.31-0.70 | 0.43 | 0.28-0.66 | 0.44 | 0.27-0.72 | 0.67 | 0.39-1.14 | 0.41 | 0.24-0.69 |
| 60-70 / BE: 60-64 | 0.81 | 0.64-1.02 | 0.54 | 0.40-0.72 | 0.52 | 0.41-0.65 | 0.41 | 0.33-0.50 | 0.59 | 0.47-0.72 | 0.90 | 0.59-1.40 | 0.44 | 0.27-0.73 | 0.50 | 0.27-0.91 | 0.72 | 0.40-1.29 | 0.29 | 0.15-0.57 |
| Nationality (ref: national citizen) |  |  |  |  |  |  |  |  |  |  |  |  |  |  |  |  |  |  |  |  |
| Foreign national | 1.66 | 1.39-1.99 | 1.76 | 1.44-2.17 | 1.75 | 1.49-2.06 | 1.58 | 1.35-1.85 | 1.5 | 1.28-1.76 | 1.48 | 1.11-1.97 | 1.34 | 0.97-1.85 | 1.47 | 1.00-2.16 | 2.17 | 1.53-3.06 | 1.47 | 0.94-2.29 |
| Area of residence (ref: urban) |  |  |  |  |  |  |  |  |  |  |  |  |  |  |  |  |  |  |  |  |
| Rural | 1.19 | 1.03-1.38 | 1.05 | 0.87-1.27 | 1.20 | 1.04-1.38 | 1.19 | 1.04-1.37 | 1.11 | 0.96-1.27 | 1.17 | 0.91-1.50 | 1.16 | 0.89-1.52 | 1.06 | 0.75-1.50 | 0.73 | 0.50-1.07 | 0.82 | 0.56-1.22 |
| Notes: APR = adjusted prevalence ratios. APR are weighted for sampling strategy in the SHIS and BHIS, and also for non-response in the SHIS. Variables used for adjustment: self-rated health, body mass index, doctor visit in the last 12 months, smoking. | | | | | | | | | | | | | | | | | | | | |
| ^a^ SHIS 1992-2012 and BHIS 1997-2013 | | | | | | | | | | | | | | | | | | | | |

| **Table S.3b. Adjusted prevalence rations (APR) for under CCS among eligible women in Switzerland and Belgium^a^** | | | | | | | | | | | | | | | | | | | | |
| --- | --- | --- | --- | --- | --- | --- | --- | --- | --- | --- | --- | --- | --- | --- | --- | --- | --- | --- | --- | --- |
|  | Under CCS in Switzerland (women aged 20-70) | | | | | | | | | | Under CCS in Belgium (women aged 25-64 y/o) | | | | | | | | | |
|  | 1992 N = 4331 | | 1997 N = 3240 | | 2002 N = 6047 | | 2007 N = 5296 | | 2012 N = 6766 | | 1997 N = 1627 | | 2001 N = 1632 | | 2004 N = 1663 | | 2008 N = 1425 | | 2013 N = 1441 | |
|  | APR | 95% CI | APR | 95% CI | APR | 95% CI | APR | 95% CI | APR | 95% CI | APR | 95% CI | APR | 95% CI | APR | 95% CI | APR | 95% CI | APR | 95% CI |
| Education (ref: primary & lower secondary) |  |  |  |  |  |  |  |  |  |  |  |  |  |  |  |  |  |  |  |  |
| Upper secondary | 0.91 | 0.75-1.10 | 0.84 | 0.64-1.09 | 0.90 | 0.72-1.13 | 0.79 | 0.62-1.01 | 1.09 | 0.87-1.35 | 0.82 | 0.53-1.29 | 0.79 | 0.55-1.13 | 0.50 | 0.36-0.71 | 1.02 | 0.72-1.47 | 1.24 | 0.85-1.81 |
| Tertiary | 0.95 | 0.71-1.26 | 0.82 | 0.51-1.33 | 0.80 | 0.57-1.11 | 0.83 | 0.62-1.13 | 1.00 | 0.77-1.30 | 0.78 | 0.48-1.26 | 0.62 | 0.40-0.98 | 0.53 | 0.37-0.76 | 1.23 | 0.82-1.85 | 0.62 | 0.38-1.01 |
| Employment (ref: employed) |  |  |  |  |  |  |  |  |  |  |  |  |  |  |  |  |  |  |  |  |
| Unemployed/ non-employed | 0.91 | 0.74-1.11 | 0.94 | 0.71-1.24 | 0.98 | 0.82-1.18 | 1.25 | 1.05-1.48 | 1.04 | 0.89-1.22 | 1.18 | 0.77-1.80 | 0.75 | 0.52-1.07 | 1.10 | 0.77-1.57 | 1.38 | 0.96-2.00 | 0.70 | 0.48-1.02 |
| Monthly household income (ref: 1st quintile) |  |  |  |  |  |  |  |  |  |  |  |  |  |  |  |  |  |  |  |  |
| 2^nd^ quintile | 0.90 | 0.68-1.18 | 1.12 | 0.83-1.52 | 0.90 | 0.71-1.15 | 1.10 | 0.85-1.42 | 1.07 | 0.88-1.31 | 0.86 | 0.49-1.51 | 0.99 | 0.62-1.58 | 1.21 | 0.790-1.85 | 0.77 | 0.48-1.22 | 0.64 | 0.40-1.02 |
| 3^rd^ quintile | 1.01 | 0.81-1.26 | 0.78 | 0.55-1.09 | 0.89 | 0.70-1.13 | 1.00 | 0.78-1.28 | 0.94 | 0.76-1.15 | 0.64 | 0.39-1.07 | 1.02 | 0.63-1.66 | 1.02 | 0.62-1.69 | 0.82 | 0.52-1.29 | 0.78 | 0.49-1.26 |
| 4^th^ quintile | 0.76 | 0.60-0.98 | 0.82 | 0.58-1.17 | 0.86 | 0.67-1.10 | 0.97 | 0.75-1.27 | 0.88 | 0.71-1.11 | 0.75 | 0.41-1.40 | 0.74 | 0.43-1.26 | 1.26 | 0.79-2.03 | 0.92 | 0.57-1.49 | 0.92 | 0.58-1.46 |
| 5^th^ quintile | 0.74 | 0.50-1.09 | 0.64 | 0.41-0.99 | 0.82 | 0.62-1.10 | 0.91 | 0.68-1.21 | 0.98 | 0.77-1.25 | 0.74 | 0.39-1.42 | 0.90 | 0.52-1.56 | 1.07 | 0.64-1.80 | 0.43 | 0.25-0.74 | 0.71 | 0.42-1.20 |
| Partnership status (ref: no partner) |  |  |  |  |  |  |  |  |  |  |  |  |  |  |  |  |  |  |  |  |
| Living with spouse/ partner | 0.98 | 0.83-1.16 | 0.70 | 0.56-0.89 | 0.87 | 0.74-1.02 | 0.82 | 0.70-0.96 | 0.81 | 0.69-0.94 | 0.87 | 0.57-1.35 | 0.94 | 0.67-1.32 | 0.70 | 0.51-0.97 | 0.94 | 0.69-1.28 | 0.86 | 0.61-1.23 |
| Age (ref: CH: 20-29/ BE: 25-29) |  |  |  |  |  |  |  |  |  |  |  |  |  |  |  |  |  |  |  |  |
| 30-39 | 1.95 | 1.22-3.11 | 1.27 | 0.79-2.06 | 1.79 | 1.02-3.13 | 1.68 | 0.98-2.88 | 1.50 | 0.99-2.26 | 2.65 | 1.01-6.98 | 1.53 | 0.65-3.63 | 2.14 | 1.07-4.26 | 4.12 | 1.56-0.88 | 0.93 | 0.39-2.24 |
| 40-49 | 3.74 | 2.38-5.86 | 2.26 | 1.41-3.63 | 2.80 | 1.61-4.87 | 3.28 | 1.99-5.41 | 2.10 | 1.45-3.03 | 2.66 | 0.99-7.21 | 2.77 | 1.21-6.36 | 2.58 | 1.31-5.06 | 5.33 | 2.05-3.90 | 1.21 | 0.52-2.82 |
| 50-59 | 6.56 | 4.19-10.27 | 2.74 | 1.68-4.45 | 3.52 | 2.04-6.10 | 4.80 | 2.91-7.92 | 3.08 | 2.15-4.42 | 4.32 | 1.54-2.11 | 3.22 | 1.36-7.63 | 2.76 | 1.37-5.53 | 8.50 | 3.29-1.98 | 1.48 | 0.64-3.42 |
| CH: 60-70/ BE: 60-64 | 10.69 | 6.83-16.74 | 6.90 | 4.31-11.04 | 5.83 | 3.36-10.12 | 6.85 | 4.17-11.25 | 4.81 | 3.36-6.89 | 5.05 | 1.76-4.69 | 5.62 | 2.22-4.23 | 5.26 | 2.54-0.89 | 9.34 | 3.44-5.41 | 3.30 | 1.42-7.71 |
| Nationality (ref: national citizen) |  |  |  |  |  |  |  |  |  |  |  |  |  |  |  |  |  |  |  |  |
| Foreign national | 1.05 | 0.80-1.37 | 0.90 | 0.64-1.27 | 0.92 | 0.70-1.23 | 0.74 | 0.53-1.04 | 0.96 | 0.76-1.22 | 1.22 | 0.65-2.27 | 1.26 | 0.67-2.39 | 0.80 | 0.40-1.60 | 0.61 | 0.35-1.07 | 1.99 | 1.17-3.38 |
| Area of residence (ref: urban) |  |  |  |  |  |  |  |  |  |  |  |  |  |  |  |  |  |  |  |  |
| Rural | 1.30 | 1.09-1.54 | 1.05 | 0.82-1.33 | 1.22 | 1.03-1.44 | 1.21 | 1.02-1.42 | 1.18 | 1.02-1.37 | 0.74 | 0.48-1.14 | 1.10 | 0.79-1.54 | 1.19 | 0.90-1.58 | 0.99 | 0.7-1.37 | 1.13 | 0.84-1.53 |
| Notes: APR = adjusted prevalence ratios. APR are weighted for sampling strategy in the SHIS and BHIS, and also for non-response in the SHIS. Variables used for adjustment: self-rated health, body mass index, doctor visit in the last 12 months, smoking. | | | | | | | | | | | | | | | | | | | | |
| ^a^ SHIS 1992-2012 and BHIS 1997-2013 | | |  |  |  |  |  |  |  |  |  |  |  |  |  |  |  |  |  |  |
